# Fibroblast growth factor23 is associated with axonal integrity and neural network architecture in the human frontal lobes

**DOI:** 10.1371/journal.pone.0203460

**Published:** 2018-09-07

**Authors:** Barbara K. Marebwa, Robert J. Adams, Gayenell S. Magwood, Mark Kindy, Janina Wilmskoetter, Myles Wolf, Leonardo Bonilha

**Affiliations:** 1 Department of Neurology, Medical University of South Carolina, Charleston, SC, United States of America; 2 Department of Nursing, Medical University of South Carolina, Charleston, SC, United States of America; 3 Department of Pharmaceutical Sciences, University of South Florida, Tampa, FL, United States of America; 4 Department of Medicine, Duke University School of Medicine, Durham, NC, United States of America; McGill University, CANADA

## Abstract

Elevated levels of FGF23 in individuals with chronic kidney disease (CKD) are associated with adverse health outcomes, such as increased mortality, large vessel disease, and reduced white matter volume, cardiovascular and cerebrovascular events. Apart from the well-known link between cardiovascular (CV) risk factors, especially diabetes and hypertension, and cerebrovascular damage, elevated FGF23 is also postulated to be associated with cerebrovascular damage independently of CKD. Elevated FGF23 predisposes to vascular calcification and is associated with vascular stiffness and endothelial dysfunction in the general population with normal renal function. These factors may lead to microangiopathic changes in the brain, cumulative ischemia, and eventually to the loss of white matter fibers. The relationship between FGF23 and brain integrity in individuals without CKD has hitherto not been investigated. In this study, we aimed to determine the association between FGF23, and white matter integrity in a cohort of 50 participants with varying degrees of CV risk burden, using high resolution structural human brain connectomes constructed from MRI diffusion images. We observed that increased FGF23 was associated with axonal loss in the frontal lobe, leading to a fragmentation of white matter network organization. This study provides the first description of the relationship between elevated levels of FGF23, white matter integrity, and brain health. We suggest a synergistic interaction of CV risk factors and FGF23 as a potentially novel determinant of brain health.

## Introduction

Fibroblast growth factor-23 (FGF23) is an osteocyte derived phosphaturic hormone that regulates calcium-phosphate and vitamin D metabolism by activating the FGF receptor-α-klotho complex in the kidney[1]. FGF23 induces phosphaturia by decreasing renal reabsorption of phosphate in the proximal tubule, and inhibiting calcitriol (hormonally active metabolite of vitamin D) synthesis[1, 2]. Calcitriol functions to increase calcium and phosphate levels in the blood by increasing kidney and gastrointestinal absorption, and increasing calcium and phosphate release from bone into the blood through bone resorption. Calcitriol inhibition by FGF23 induces calcium deficiency resulting in even more production of calcium from the bone. Excess circulating calcium eventually leads to arterial and vascular calcification[3]. Chronic Kidney Disease (CKD) is possibly the most common cause of elevated FGF23, which has been implicated in increased cardiovascular mortality of CKD patients. Elevated FGF23 is also associated with cardiovascular disease, left ventricular hypertrophy [4, 5], and is a putative indicator of Cardiovascular (CV) risk factors [6]. Apart from CKD, high phosphorous diet stimulates FGF23 production, leading to elevated levels of FGF23 [7]. Even among individuals without CKD, elevated levels of FGF23 have been postulated to increase the risk of stroke [8]. Nonetheless, the effects of elevated FGF23 on brain health in non-stroke individuals have not fully been determined. Compared with brain gray matter, white matter is significantly more susceptible to small vessel ischemic injury because it receives less perfusion when adjusting for metabolic demands, due to lower collateral blood supply to deep white matter [9]. Moreover, the maintenance of structural integrity of medium to long range axonal projections is metabolically demanding. For these reasons, we postulated that FGF23 would lead to white matter compromise particularly within the brain areas with long cortico-cortical, or cortico-subcortical axonal projections, such as the frontal lobes.

We therefore aimed to identify the effects of FGF23 independent of kidney disease by studying a prospective cohort with normal kidney function, but with CV risk factors that included diabetes, hypertension, and hyperlipidemia. We employed the novel neuroimaging method of the high-resolution human brain connectome to fully map white matter networks across the entire brain. We aimed to determine the relationship between FGF23 and neuronal network integrity, with the goal of elucidating the mechanistic aspects related to the impact of FGF23 on brain health. We postulated a synergistic interaction of CV risk factors and FGF23 as a determinant of brain health.

## Methods

### Participants

We recruited 51 older participants, (40 females, mean age 55.3 ± 8.6 years) without a history of neurological or psychiatric diseases from the local community through advertisement. All participants were self-reported cognitively normal adults. There were 23 African American and 28 white participants. Twenty-eight participants did not have a history of cardiovascular risk factors, while 23 participants had previously been diagnosed with at least one CV risk factor (CV group): diabetes (11 participants), hyperlipidemia (15 participants), and hypertension (16 participants). Six participants had been diagnosed with all CV risk factors. These diagnoses were obtained through medical chart review. The Charlson Comorbidity Index[10] (CCI) was calculated for all participants, including a diagnosis of hypertension and hyperlipidemia at a score of one each to the overall score. BMI and smoking history were not available for all participants and therefore not included in the analyses. Participants were stratified into two groups: CV risk factor and normal controls based on a previous diagnosis of a cardiovascular disease. One participant had chronic kidney disease and was therefore excluded from further analysis. All participants included in the analysis had normal renal function. The study was approved by the Institutional Review Boards at the Medical University of South Carolina. Written informed consent was obtained from all participants, as approved by our institutions’ IRB.

### FGF23 acquisition

Circulating FGF23 was measured using the Human FGF23 ELISA kit from Millipore (EZHFGF23-32K). Samples were collected in EDTA containing tubes and centrifuged at 2-3K to obtain the plasma. Samples were prepared as described by the manufacturer and the concentrations of FGF23 were determined from the standards provided. FGF23 is presented as pg/ml of plasma.

### Image acquisition

Imaging was performed on a Siemens 3T TIM trio MRI scanner located at the Medical University of South Carolina. We used volumetric T1-weighted and Diffusion images collected from each participant. T1 parameters: MPRAGE sequence with 1 mm isotropic voxels, 256x256 matrix size, and a 9-degree flip angle. We used a 192-slice sequence with TR = 2250 ms, T1 = 925 ms, and TE = 4.11 ms. DTI parameters: twice-refocused echo-planar imaging b = 0, 1000, 30 diffusion encoding directions, TR = 8500 ms, TE = 98 ms, FOV = 222 x 222 mm2, matrix = 74 x 74, 3 mm slice thickness, and 40 axial slices.

### Structural connectome construction

Each participant’s individual high-resolution structural connectome was built from structural T1 and diffusion tensor imaging (DTI) neuroimaging data using the following steps: 1.T1 weighted images were spatially registered into standard space and segmented into probabilistic grey and white matter maps using SPM12’s unified segmentation-normalization; 2.Each individual’s grey matter map was divided into 1358 approximately evenly sized regions using the Atlas of Intrinsic Connectivity of Homotopic Areas (AICHA) brain atlas [11]; 3.The grey matter parcellation maps were then non-linearly registered into the diffusion imaging (DTI) space, and pairwise probabilistic DTI fiber tracking was computed for all possible pairs of grey matter regions 4. The weight of each pairwise connectivity link was determined based on the number of probabilistic streamlines connecting the grey matter region pair, corrected by distance travelled by each streamline and by the total volume of the connected regions. Finally, a weighted adjacency matrix M of size 1358 x 1358 was constructed for each participant with M_*i*,*j*_ representing the weighted link between region of interest (ROI) *i* and ROI *j*. Tractography was estimated through the software FSL FMRIB's Diffusion Toolbox (FDT), including eddy current correction, motion correction[12], and probabilistic method [13] with BEDPOST being used to assess default distributions of diffusion parameters at each voxel, and probabilistic tractography was performed using FDT’s probtrackX (parameters: 5000 individual pathways drawn through the probability distributions on principal fiber direction, curvature threshold set at 0.2, 200 maximum steps, step length 0.5mm, and distance correction). The weighted connectivity between the regions *i* and *j* was defined as the number of probabilistic streamlines arriving at *j* region when *i* was seeded, averaged with the number of probabilistic streamlines arriving at *i* region when *j* was seeded.

**Fig 1:**
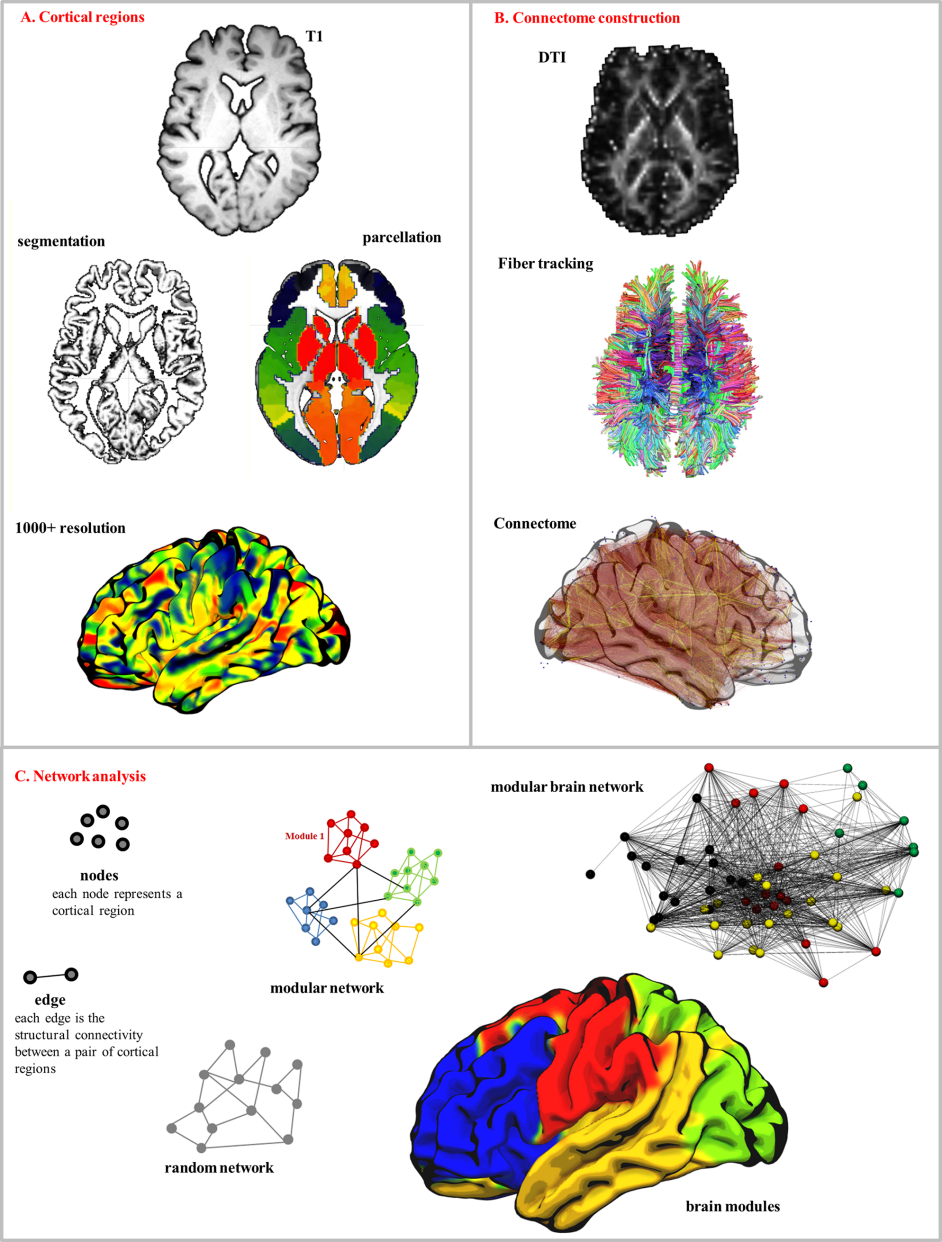
Connectome generation and network analysis. In A, the T1 image is normalized, and segmented (into CSF, gray and white matter). The gray matter is parcellated into 1358 regions of interest (ROI). The T1 is warped into diffusion space where fiber tracking occurs, finding the connections between each pair of ROIs, generating a connectome, or network of connectivity between all brain regions. C is an example of whole brain modular partition into modules using Newman’s modularity algorithm, which groups ROIs that are more closely associated by their white matter networks and relatively segregated from surrounding groups (each module is represented by the same color).

Fig 1 provides a workflow of the connectome construction process and network analysis.

### Modular organization detection

The integrity of neuronal network architecture can be assessed through the quantification of the modular parcellation of the network (modularity). Modularity provides a measurement of the balance between segregation and integration of the network in its entirety, or within regional sub-networks. This balance is known to be a fundamental principle in biological network organization, including neuronal networks [14]. Our analysis was based on the modular organization of each brain region for every participant. For each participant the whole brain connectome was divided into the left and right frontal, temporal, parietal, and occipital lobe sub-networks. The lobe sub-networks were assessed regarding their modularity using Newman’s modularity algorithm [15] implemented in the Brain Connectivity Toolbox [16], (e.g. [Ci,Q] = modularity_und(W), where W is the weighted undirected connectivity matrix; gamma was maintained at the default: gamma = 1).

### Statistical analyses

For each group (CV risk factor and healthy controls), we performed general linear regression analysis to examine the relationship between brain integrity and FGF23, adjusting for key covariates–sex, race, and CCI. Linear regressions were modelled for each group separately to minimize noise inherent in the healthy control group, and to avoid type II error. Brain integrity measured via modularity was set as the dependent variable, and FGF23, sex, race and CCI as the predictor variables. We used this model to determine the association between FGF23, and the integrity of each brain region (modularity scores for the frontal, temporal, parietal and occipital regions) for both left and right hemispheres. Linear correlations were evaluated using a two-tailed Pearson correlation coefficient. We evaluated the association between FGF23 and modularity of the left and right frontal hemispheres. Correlation was performed independently on the CV risk factor group and the control group.

We further assessed the overall connectivity of the frontal lobe by calculating the density; a measure of axonal integrity; as the number of all connections present. We then determined the relationship between FGF23 and fiber density of the left and right hemisphere frontal regions.

Of the 22 participants with CV risk factors, 17 had serum creatinine scores from the latest available comprehensive metabolic panel obtained from the hospital. We re-calculated the Pearson correlation for these participants, with partial correlations accounting for the creatinine score. Natural log transformed FGF23 scores were used in all analyses. All statistical analyses were performed using MATLAB. The statistical significance was set at p ≤ 0.05.

## Results

To account for possible confounding by age, sex and race, we determined the association between these factors and FGF23. Pearson analysis revealed no correlation between age and FGF23 (r = 0.13, p = 0.17), and student’s t-test revealed no differences in FGF23 levels between female and male participants two-sample t(48) = 0.91, p = 0.37, or between African American and Caucasian participants two-sample t(48) = 0.53, p = 0.6. There was also no significant difference in FGF23 levels between the control and CV risk factor groups, two-sample t(48) = 0.69, p = 0.49.

### Relationship between modularity and FGF23

Our model revealed that FGF23 was associated with left hemisphere frontal lobe modularity in the CV risk factor group: F(4,22) = 4.2, p = 0.015, adjusted R^2^ = 0.38 (Table 1). FGF23 was not associated with brain integrity among individuals without CV risk factors. In that group, brain integrity was associated with sex, race and CCI (Table 2). A model of only FGF23 (predictor variable) and left hemisphere frontal lobe modularity (dependent variable) revealed that FGF23 levels alone accounted for about 29% of brain integrity in the left hemisphere frontal lobe of participants with CV risk factors: F(1,22) = 9.46, p = 0.006, adjusted R^2^ = 0.29, while the same was not observed in the control group: F(1,22) = 0.6, p = 0.45, adjusted R^2^ = 0.015.

**Table 1:** Multiple linear regression models for modularity in participants with cardiovascular risk factors.

Outcome		Model	Variables			
			FGF23	Gender Female vs male	Race White vs black	CCI
LH frontal						
	Adj.R^2^ = 0.38	B (SE)	0.03 (0.01)	-0.06 (0.03)	-0.01 (0.03)	0.02 (0.01)
	F = 4.20	β	0.52*	-0.43*	-0.10	0.31
RH frontal						
	Adj.R^2^ = 0.21	B (SE)	0.02 (0.02)	-0.05 (0.04)	0.01 (0.04)	0.04 (0.02)
	F = 2.40	β	0.17	-0.26	0.05	0.55*
LH parietal						
	Adj.R^2^ = 0.12	B (SE)	-0.02 (0.02)	-0.01 (0.04)	0.09 (0.04)	-0.01 (0.02)
	F = 1.72	β	-0.25	-0.07	0.57*	-0.10
RH parietal						
	Adj.R^2^ = 0.03	B (SE)	-0.01 (0.02)	-0.05 (0.03)	0.03 (0.03)	0.01 (0.01)
	F = 1.19	β	-0.18	-0.36	0.23	0.14
LH temporal						
	Adj.R^2^ = -0.12	B (SE)	-0.00 (0.01)	-0.02 (0.02)	-0.01 (0.02)	0.01 (0.01)
	F = 0.44	β	-0.07	-0.22	-0.10	0.30
RH temporal						
	Adj.R^2^ = 0.12	B (SE)	0.01 (0.01)	-0.05 (0.03)	0.01 (0.03)	0.01 (0.03)
	F = 1.70	β	0.11	-0.44	0.04	0.34
LH occipital						
	Adj.R^2^ = 0.22	B (SE)	-0.01 (0.01)	-0.02 (0.03)	0.07 (0.03)	0.01 (0.01)
	F = 2.49	β	-0.21	-0.18	0.55*	0.10
RH occipital						
	Adj.R^2^ = -0.10	B (SE)	0.01 (0.01)	-0.01 (0.03)	0.02 (0.03)	0.01 (0.01)
	F = 0.51	β	0.12	-0.07	0.20	0.12

*p < .05.

**p < .01.

B = parameter estimate, SE = standard error, β = standardized estimate, CCI = Charlson Comorbidity Index, LH = left hemisphere, RH = right hemisphere

**Table 2:** Multiple linear regression models for modularity in healthy controls.

Outcome		Model	Variables			
			FGF23	Gender Female vs male	Race White vs black	CCI
LH frontal						
	Adj.R^2^ = 0.38	B (SE)	0.03 (0.01)	-0.06 (0.03)	-0.01 (0.03)	0.02 (0.01)
	F = 4.20	β	0.52	-0.43	-0.10	0.31
RH frontal						
	Adj.R^2^ = 0.32	B (SE)	0.02 (0.01)	0.08 (0.04)	0.10 (0.03)	0.02 (0.01)
	F = 4.12	β	0.17	0.29	0.62**	0.20
LH parietal						
	Adj.R^2^ = 0.23	B (SE)	-0.01 (0.02)	0.10 (0.06)	0.11 (0.04)	0.03 (0.02)
	F = 2.97	β	-0.04	0.31	0.52**	0.27
RH parietal						
	Adj.R^2^ = 0.15	B (SE)	0.01 (0.02)	0.12 (0.07)	0.11 (0.04)	0.03 (0.02)
	F = 2.21	β	0.06	0.32	0.47	0.22
LH temporal						
	Adj.R^2^ = 0.49	B (SE)	0.02 (0.01)	0.02 (0.03)	0.08 (0.02)	0.04 (0.01)
	F = 7.48	β	0.20	0.08	0.57**	0.50**
RH temporal						
	Adj.R^2^ = 0.06	B (SE)	0.01 (0.01)	0.01 (0.04)	0.05 (0.02)	0.01 (0.01)
	F = 1.45	β	0.18	0.07	0.40	0.18
LH occipital						
	Adj.R^2^ = 0.31	B (SE)	-0.01 (0.01)	0.06 (0.03)	0.03 (0.02)	0.03 (0.01)
	F = 4.08	β	-0.20	0.37*	0.24	0.49**
RH occipital						
	Adj.R^2^ = 0.23	B (SE)	0.01 (0.01)	-0.02 (0.03)	0.05 (0.02)	0.02 (0.01)
	F = 3.07	β	0.16	-0.12	0.47*	0.24

*p < .05.

**p < .01.

B = parameter estimate, SE = standard error, β = standardized estimate, CCI = Charlson Comorbidity Index, LH = left hemisphere, RH = right hemisphere

**Fig 2:**
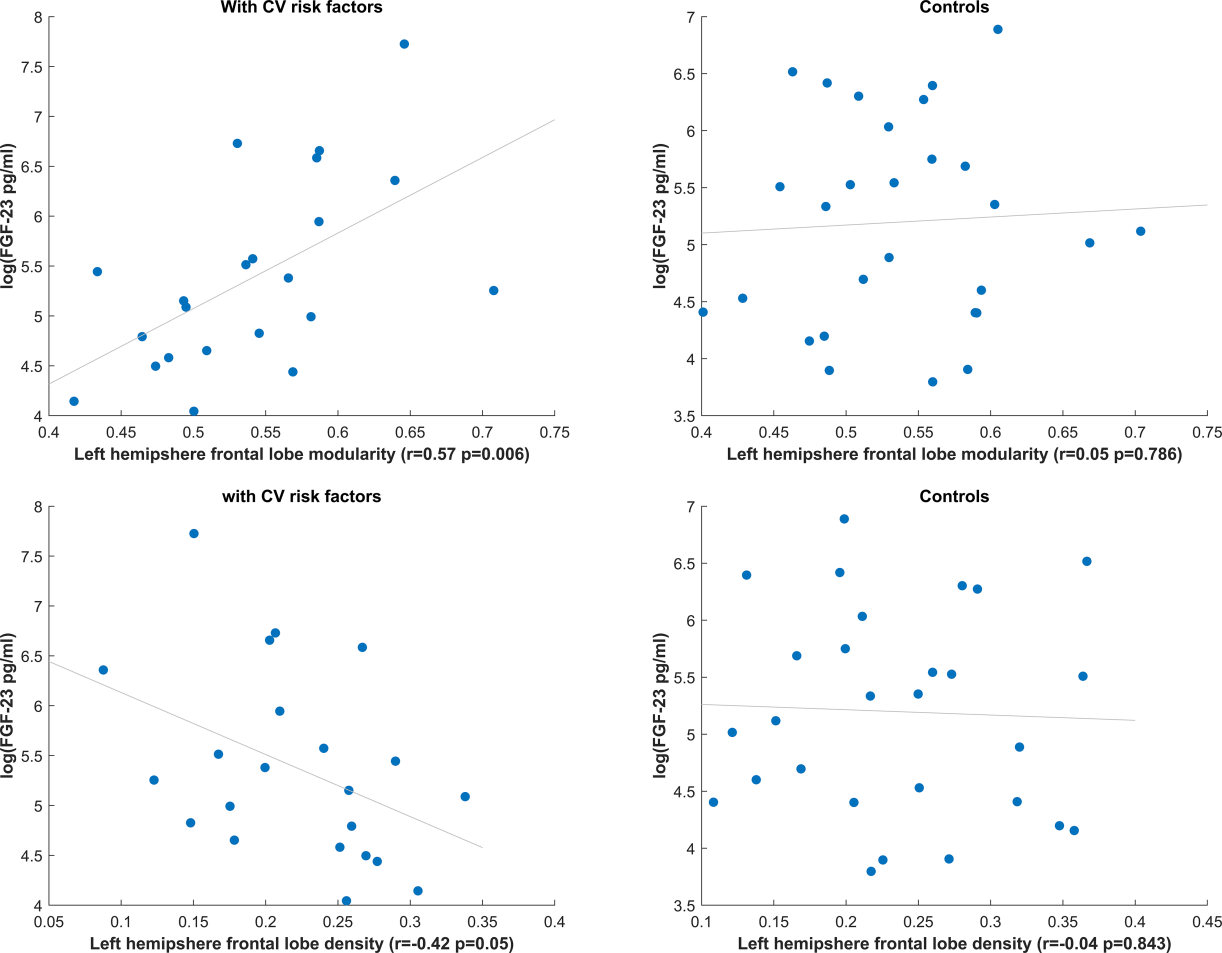
**A)** Left panel–correlation between left hemisphere frontal lobe modularity and FGF23 in the CV risk factor group. Right panel–correlation between left hemisphere frontal lobe modularity and FGF23 in the control group. B) Left panel—correlation between left hemisphere frontal lobe density and FGF23 in the CV risk factor group. Right panel–correlation between left hemisphere frontal lobe density and FGF23 in the control group.

Linear correlations revealed that left hemisphere frontal lobe modularity was significantly correlated with FGF23 (Fig 2A left panel) in participants with CV risk factors, such that higher modularity was associated with higher FGF23 levels (r = 0.57, p = 0.006). This association was also significant when controlling for creatinine levels (n = 17) (partial correlation controlling for creatinine: r = 0.48, p = 0.03). In the control group, the left hemisphere frontal lobe modularity was not associated with FGF23 levels (Fig 2A right panel, r = 0.05, p = 0.4).

The right hemisphere frontal lobe was not associated with FGF23 in the CV risk factor group (r = 0.22, p = 0.33) or control group (r = 0.12, p = 0.55). Correcting for multiple comparisons (0.05/8), the left hemisphere frontal lobe modularity was still significantly correlated with FGF23.

### Relationship between density and FGF23

#### Frontal lobe

There was a significant correlation between the left frontal lobe density and FGF23 (Fig 2B left panel) in participants with CV risk factors such that participants with elevated FGF23 had fewer fiber connections in their left frontal lobe (r = -0.42, p = 0.05). We further calculated correlation on 17 participants with creatinine scores (partial correlation controlling for creatinine: r = -0.40, p = 0.07). An association was not observed in the left frontal hemisphere of participants without CV risk factors (r = -0.04, p = 0.58, Fig 2B right panel).

## Discussion

In this study, we aimed to determine the relationship between CV risk factors, FGF23 levels, integrity of axonal fibers, and white matter network topological organization. We employed high resolution structural connectomes constructed from diffusion imaging to measure individual network integration and segregation via modularity. We hypothesized that elevated FGF23 levels would be associated with the reduction of white matter fiber connections, and loss of organization (fragmentation) of white matter networks. We demonstrated a relationship between FGF23 and left frontal lobe network integrity, and showed that in participants with CV risk factors, FGF23 levels accounted for up to 29% of left hemisphere frontal lobe network integrity, meaning that participants with elevated FGF23 levels and CV risk factor burden had higher modularity scores, indicating a disruption of the brain network organization. Sex, race, and CCI explained a further 9% of left frontal lobe integrity. FGF23 was not significantly associated with brain integrity in participants without CV risk factors, although race, sex and CCI were significantly associated with brain integrity in this group.

Our results complement and help explain previous findings suggesting that elevated FGF23 is associated with stroke and small vessel disease (SVD) independent of CKD. SVD is routinely observed in normal ageing or individuals with CV risk factors and discovered incidentally on routine MRIs by white matter hyperintensities (WMH). The Northern Manhattan study (NOMAS) prospectively assessed 2,525 individuals from a racially diverse population and concluded that elevated FGF23 conferred an overall risk of stroke and intracerebral hemorrhage independent of CKD[8]. In a subset of the same cohort, (n = 1170), they also showed that elevated FGF23 was associated with WMH, demonstrating a link between FGF23 and SVD in the absence of CKD[17]. The Professional Follow-up Study (n = 1261) further showed elevated FGF23 in individuals with established CV risk factors and higher dietary phosphate intake.

Our study builds on this previous literature by employing an approach that quantifies network integrity; and may detect subclinical structural compromise; to assess the tripartite association between CV risk factors, FGF23, and white matter integrity. We employed a fine-grained atlas to improve our statistical power, and for a more detailed connectome; we did not assess the effect of using functionally relevant atlases, although whole brain tractography did yield functionally relevant modules (Fig 1 panel C)

We observed reduced network density, and a disorganization of the left frontal hemisphere network topology related to elevated FGF23, exclusively in participants with CV risk factors. Importantly, we showed that FGF23 is an independently associated with brain integrity in participants diagnosed with CV risk factors. Since FGF23 is a modifiable risk factor, understanding this association may be an important step in reducing stroke incidences, stroke severity, and improving outcome after stroke. Brain integrity of normal ageing individuals was still explained by age and comorbidity (captured by CCI), sex, and race, although with the current limited sample size, we cannot conclusively draw any conclusions on the significance of sex and race.

The frontal hemisphere supports several cognitive processes including problem solving, memory, language, judgement, social behavior and impulse control. Therefore, the association between FGF23 and compromised structural integrity of the frontal lobe further provides an interesting first step towards understanding the impact of FGF23 on cognition. The fiber loss localized to the left hemisphere, indicating that the relationship between FGF23 and network fragmentation is restricted to the dominant hemisphere (all participants except 1in the CV group were right handed). This observation may indicate a more pronounced susceptibility to injury in the dominant hemisphere.

The limitations of this study are: 1) we did not assess the association between white matter network integrity and behavioral function since a comprehensive assessment of neuropsychological performance was not available in this cohort. We believe that this would be an important future direction, specifically testing the impact of FGF23 on cognitive control and executive function, which are frontal lobe dependent measures. 2) This is an initial pilot study with a relatively small sample size. Furthermore, the multiple linear regression models were not controlled for multiple comparisons, even though we did account for multiple confounders. 3) BMI and smoking history are important CV risk factors that were not included in the analyses. 4) Our cohort is made up of 80% women and is therefore not a not representative sample of the general population.

Based on the findings of this study, we believe that there are important possible future directions to continue to elucidate the impact of FGF23 on brain health, namely 1) the assessment of the cognitive impact of FGF23, and whether FGF23 leads to subclinical yet quantifiable cognitive compromise, particularly in frontal lobe functions; 2) the evaluation of the continuum between the impact of FGF23 on brain health across the spectrum ranging from normal kidney function to end-state CKD; 3) the assessment of the synergistic effects of FGF23 with other CV, electrolyte and kidney function biomarkers; and 4) the concurrent evaluation of connectome with other measures of brain integrity, including T2 weighted microangiopathic lesion burden.

In summary, we demonstrated the association between elevated serum FGF23, fiber loss and network disintegration in the frontal region of the left hemisphere. This relationship between FGF23 and brain integrity was noted in individuals without CKD, but with CV risk factors. We postulate a synergistic interaction of CV risk factors and FGF23 as a potentially novel determinant of brain health.
